# Supplementary material for: Membrane Targeting and GTPase Activity of Rab7 Are Required for Its Ubiquitination by RNF167
Source: Int J Mol Sci. 2022 Jul 16;23(14):7847. doi: 10.3390/ijms23147847 (PMC9319455; doi:10.3390/ijms23147847)
Supplement: Supplementary file 1 [file ijms-23-07847-s001.zip › ijms-1806781-supplementary.pdf]

Supplementary material (Python code)

Membrane Targeting and GTPase Activity of Rab7 Are Required for Its Ubiquitination by RNF167

Kim Ghilarducci [1,2], Valérie C. Cabana [1,2], Ali Harake [1,2], Laurent Cappadocia [1,2] and Marc P. Lussier [1,2,*]

[1] Département de Chimie, Université du Québec à Montréal, Montréal, QC H2X 2J6, Canada; ghilarducci.kim@courrier.uqam.ca (K.G.); cabana.valerie@courrier.uqam.ca (V.C.C.); harake.ali@courrier.uqam.ca (A.H.); cappadocia.laurent@uqam.ca (L.C.)

[2] Centre d'Excellence en Recherche sur les Maladies Orphelines-Fondation Courtois (CERMO-FC), Université du Québec à Montréal, Montréal, QC H2X 3Y7, Canada

* Correspondence: lussier.marc@uqam.ca; Tel.: +1-514-987-3000 (ext. 5591); Fax: +1-514-987-4054

Supplementary material (Python code)

```
# This code, written in Python 3, is used to analyze Lamp1 distribution. Prior to using the code,
# ImageJ is used to extract Lamp1 fluorescence signal over a line extending from the edge of the
# nucleus (detected using the DAPI signal) to the plasma membrane (detected using the Lamp1 signal).
# Line plots are then exported from ImageJ in csv file format and csv files are successively read and
# analyzed using the following script. The script basically divides the line plot into 10 segments and
# returns the ratio between the average fluorescence signal of segments 2 and 9.

import pandas as pd
import numpy as np

#File selection
myfile = pd.read_csv("002-line-1.csv")

intensities = myfile["Gray_Value"]
backgroundSubstratedIntensities=intensities-min(intensities)

#Selection of the segment close to nucleus
s2 = np.array_split(backgroundSubstratedIntensities, 10) [1]

#Selection of the segment close to plasma membrane
s9 = np.array_split(backgroundSubstratedIntensities, 10) [8]

print("The average intensity of segments 2 and 9 is %.2f and %.2f, respectively" % (np.average(s2),
np.average(s9)))
print("The intensity ratio between segments 2 and 9 is %.3f" % (np.average(s2) / np.average(s9)))

# Script output:
#
# The average intensity of segments 2 and 9 is 647.22 and 1431.30, respectively
# The intensity ratio between segments 2 and 9 is 0.452

# Content of the file "002-line-1.csv":
#
# Distance_(microns),Gray_Value
# 0.0000,253.000
# 0.1083,762.280
# 0.2167,1113.436
# 0.3250,1266.380
# 0.4333,2074.948
# ...
```
